# Expanding and testing fluorescent amplified fragment length polymorphisms for identifying roots of boreal forest plant species

**DOI:** 10.1002/aps3.1236

**Published:** 2019-04-08

**Authors:** Paul Metzler, Marc La Flèche, Justine Karst

**Affiliations:** ^1^ Department of Renewable Resources University of Alberta Edmonton T6G 2E3 Alberta Canada

**Keywords:** boreal forest, fluorescent amplified fragment length polymorphisms, plant identification, root cores, Sanger sequencing, soil cores

## Abstract

**Premise of the Study:**

Identifying roots to species is challenging, but is a common problem in ecology. Fluorescent amplified fragment length polymorphisms (FAFLPs) can distinguish species within a mixed sample, are high throughput, and are inexpensive. To broaden the use of this tool across ecosystems, unique size profiles must be established for species, and its limits identified.

**Methods:**

Fragments of three noncoding cpDNA regions were used to create size profiles for 193 species common to the western Canadian boreal forest. We compared detection success among congeners using FAFLPs and Sanger sequencing of the *trnL* intron. We also simulated and experimentally created communities to test the influence of species richness, cpDNA regions used, and extraction/amplification biases on detection success.

**Results:**

Of the 193 species, 54% had unique size profiles. This value decreased when fewer cpDNA regions were used. In simulated communities, ambiguous species identifications were positively related to the species richness of the community. In mock communities, some species evaded detection owing to poor extraction or amplification. Sequencing did not increase detection success compared to FAFLPs for a subset of 24 species across nine genera.

**Discussion:**

We recommend FAFLPs are best suited to confirm rather than discover species occurring belowground.

Plant identification is fundamental to ecology. Although identifying flowers and leaves is relatively straightforward, identifying roots to species can be difficult. Identification is especially challenging when roots are excised from aboveground stems and sampled in bulk, i.e., the usual method of sampling roots with a soil core. With this sampling approach, users require identification tools that distinguish species within a mixed sample, and are high throughput and inexpensive. While we focus on this problem for roots, the issue is broadly applicable to any study where mixed‐species samples composed of plant fragments must be effectively and quickly identified (e.g., forensics, diet contents, paleobotany, eDNA).

Of the current tools, molecular approaches have been identified as effective and reliable methods to identify roots to species (Rewald et al., 2012) (Table 1). For instance, species‐specific primers that amplify DNA of target species have been used to amplify fragments of distinct sizes characteristic of grassland (McNickle et al., 2008) and forest plant species (Zeng et al., 2017). However, creating species‐specific primers may be impractical because sequence information is not readily available for many plant species, primers take time to test and optimize, and there is a limit to how many unique species‐specific size profiles can be created for a DNA region that is only a few hundred base pairs long. Next‐generation sequencing can generate thousands of relatively short DNA sequences from multiple species present in a sample (e.g., Lamb et al., 2016), reducing the number of extractions required. This level of sequencing, however, may be superfluous when identifying roots excised within a core, where we expect species richness to be relatively low (e.g., Frank et al., 2010). The short DNA sequences produced by next‐generation sequencing may also limit species identification, necessitating amplification of multiple regions.

**Table 1:** Comparison of current molecular methods available to identify roots to species

Method	Product	Multiplexing	Relative costs				Ability to capture species richness in root community
			DNA extraction/unit of extractions	PCR reagents	Sequencing (96 samples)	Bioinformatics	
Designed primers to target individual species	DNA fragments	No	High/mixed roots	Low	None	None	Low
Sanger sequencing	DNA sequences (long, ~800 bp)	No	Low/individual root	Medium–high (BigDye Terminator sequencing reagent required)	Low	None if <200 samples; Yes if >200 samples	High, with multiple markers
Next‐generation sequencing	DNA sequences (short, ~300 bp)	Yes	High/mixed roots	Medium–high (high fidelity *Taq* polymerase required, and platform‐specific adapters for multiplexing)	High (platform‐specific sequencing reagents required)	Yes	High, with multiple markers
FAFLPs	DNA fragments	Yes	High/mixed roots	Low	Low	None	Low

Note

FAFLP = fluorescent amplified fragment length polymorphism.

Other candidate tools include first‐generation sequencing, which generates a DNA sequence from an individual organism, and size‐based markers such as fluorescent amplified fragment length polymorphisms (FAFLPs). Using FAFLPs, size profiles from fluorescently labeled PCR amplicons (a fragment of DNA produced by PCR) derived from unknown roots are compared to those developed from known species (Ridgway et al., 2003; Taggart et al., 2011; Randall et al., 2014). Multiple regions of DNA are amplified to increase the likelihood of identifying unique polymorphisms because there is no single gene barcode to identify plant species (Hollingsworth et al., 2011). Fragment lengths have correctly identified species in mixed samples (Ridgway et al., 2003; Moore and Field, 2005) of up to 16 species (Taggart et al., 2011). In particular, FAFLP size keys have been previously developed for plants of two common ecosystems in western Canada, aspen parkland (Taggart et al., 2011) and the boreal forest (Randall et al., 2014).

One known issue with FAFLPs, however, is the inability to distinguish among some closely related species (Ridgway et al., 2003; Taggart et al., 2011; Randall et al., 2014). Sanger sequencing generates data of higher resolution than that derived from fragment lengths of a given amplified region, and as such, sequences may be more effective to differentiate congeners than FAFLPs. Although DNA can be extracted from bulk roots for FAFLPs, it must be separately extracted from each root fragment for Sanger sequencing (e.g., Kesanakurti et al., 2011), adding considerable time to the latter method (single vs. multiple extractions). Additional costs for Sanger sequencing arise in the actual sequencing step, which is otherwise unnecessary in FAFLPs because it is a size‐based technique. Thus, DNA sequences potentially provide higher resolution to species identification, but may do so at a higher cost.

Toward the goal of increasing our ability to identify excised roots from soil cores, we first expanded reference fragment size profiles for plants in the western Canadian boreal forest. To accomplish this, 209 species were collected and analyzed for FAFLPs using three cpDNA regions (the *trnT*‐*trnL* intergenic spacer, the *trnL* intron, and the *trnL*‐*trnF* intergenic spacer) to generate a size key for identifying roots to species. Under this first objective, we doubled the number of species serving as references for future studies involving species identification of roots, and we created a searchable database. Our second objective was to identify limitations of FAFLPs in species identifications. Toward this next goal, we first compared detection success for a subset of congeners identified by FAFLPs and Sanger sequencing. Next, we used simulated and experimental mock communities to test how species richness and cpDNA regions used as species markers influence detection success. We conclude that FAFLPs are best suited for conditions of low plant diversity and where the sampled species pools are constrained by species known to occur aboveground.

## METHODS

### Field collection of reference plant tissue: Leaves

Leaves were collected from one to six individuals from 209 species common to the boreal forest of western Canada (Johnson et al., 1995). Sixty 30 × 30‐m plots were chosen to represent a range of natural and disturbed habitats from across the region. These plots were searched intensively (time unlimited) by walking 15 transects, each the length of the plot, and checking for new species within one meter of the transects. Sites included jack pine (*Pinus banksiana* Lamb.), white spruce (*Picea glauca* (Moench) Voss), black spruce (*Picea mariana* (Mill.) Britton, Sterns & Poggenb.), aspen (*Populus tremuloides* Michx.), and mixedwood‐dominated upland sites, forested wetlands, and disturbed sites, such as abandoned oil and gas well pads and roadsides. Sampling covered an approximately 30,000‐km^2^ region from 56°0′21.49″N to 54°32′27.73″N latitude (NAD 83). Replicates of the same species were taken from different plots separated by at least 25 m to capture intraspecific genetic variation.

Approximately 20 g of disease‐free leaves, showing no signs of herbivory or infection, were collected for each sample in paper bags and kept on ice until frozen (−20°C) at the end of the day. For smaller herbs, stems were collected as well. For each species, a voucher specimen was collected, mounted, and deposited at the University of Alberta Herbarium (UAPC ALTA‐VP COLLECTION 140869–141088). Frozen samples were thawed and washed with deionized water and left to air‐dry until excess moisture was removed. Aluminum packets were folded around the plant samples and lyophilized using a benchtop freeze dryer (FreeZone 2.5 Liter Benchtop Freeze Dry System; Labconco Corporation, Kansas City, Missouri, USA) for three to four days. Using sterilized forceps, approximately 40 mg of plant material was placed in a 2‐mL tube along with three sterilized 3‐mm tungsten carbide beads. Samples were tissue‐lysed on a TissueLyser II (QIAGEN, Hilden, Germany) for 2 min at 30 rotations per second, repeated if necessary until pulverized.

### Determining fragment size profiles for species

In total, FAFLP analysis was run on 2040 samples (680 individuals × three cpDNA regions). Consistent with our goal to keep costs low, two “homebrew” DNA extraction methods were used instead of proprietary DNA extraction kits. Specifically, total genomic DNA of leaves was extracted based on a modified 2% hexadecyltrimethylammonium bromide (CTAB) protocol (Roe et al., 2010). Using this extraction method, only 44% of samples produced fragment lengths. Specifically, success rates for the *trnL* intron, the *trnT*‐*trnL* intergenic spacer, and the *trnL*‐*trnF* intergenic spacers were 30%, 55%, and 47%, respectively. Owing to the low success, DNA was re‐extracted for common species for which one or more regions were unresolved using a second method, 5% CTAB and a polyethylene glycol (PEG) precipitation (Griffiths et al., 2001). The FAFLP analysis was run on 422 samples re‐extracted with this new method, of which 61% produced fragment lengths. Success rates for the *trnL* intron, the *trnT*‐*trnL* intergenic spacer, and the *trnL*‐*trnF* intergenic spacers were higher: 54%, 67%, and 68%, respectively, even though these samples were nonrandomly chosen from a group more likely to fail (i.e., from samples that were unsuccessful using the 2% CTAB method).

Of the 15 species for which no fragments were recovered, six (*Cinna latifolia* (Trevir. ex Göpp.) Griseb., *Equisetum fluviatile* L., *Gymnocarpium dryopteris* (L.) Newman, *Senecio eremophilus* Richardson, *Symphyotrichum puniceum* (L.) Á. Löve & D. Löve var. *puniceum*, and *Vaccinium cespitosum* Michx.) were not recovered by either extraction method, and nine (*Campanula rapunculoides* L., *Geranium bicknellii* Britton, *Geum rivale* L., *Juncus bufonius* L., *Juniperus horizontalis* Moench, *Lathyrus venosus* Muhl. ex Willd., *Lonicera villosa* (Michx.) Schult., *Maianthemum trifolium* (L.) Sloboda, and *Malaxis monophyllos* (L.) Sw.) were not tried with the second extraction method because they are less common in the boreal forest of Alberta.

Three regions were targeted with the universal primer sets established by Taberlet et al. (1991): the *trnT*‐*trnL* intergenic spacer, the *trnL* intron, and the *trnL*‐*trnF* intergenic spacer with a modified *trnT*‐*trnL* forward primer (Cronn et al., 2002) (Table 2). Forward primers in each primer pair were fluorescently labeled (A2: FAM, C: VIC, E: NED; Integrated DNA Technologies, Coralville, Iowa, USA). PCRs were carried out in 25‐μL volumes: 12.5 μL of EconoTaq PLUS 2× Master Mix (Lucigen Corporation, Middleton, Wisconsin, USA), 2.5 μL of each forward and reverse primer at 10 μM, 5.5 μL of autoclaved deionized water, and 2 μL of 5–10 ng·μL^−1^ DNA template. Negative controls to account for contamination during amplification were included for each 96‐well plate (no contamination was observed). Reactions were performed using an Eppendorf Mastercycler Pro S gradient thermal cycler (model 6321; Eppendorf Canada, Mississauga, Ontario, Canada). Reaction conditions were the same for all three regions, slightly modified from Taggart et al. (2011): 94°C for 5 min; followed by 35 cycles of 94°C for 60 s, 60°C for 60 s, 72°C for 80 s; and a final extension of 72°C for 30 min.

**Table 2:** Primers used to isolate three cpDNA regions in this study: the *trnT*‐*trnL* intergenic spacer, the *trnL* intron, and the *trnL*‐*trnF* intergenic spacer.^a^

Region	Name	Primer sequence (5′–3′)
*trnT*‐*trnL*	A2	F: CAAATGCGATGCTCTAACCT
	B	R: TCTACCGATTTCGCCATATC
*trnL*	C	F: CGAAATCGGTAGACGCTACG
	D	R: GGGGATAGAGGGACTTGAAC
*trnL*‐*trnF*	E	F: GGTTCAAGTCCCTCTATCCC
	F	R: ATTTGAACTGGTGACACGAG

a These universal primer sets were established by Taberlet et al. (1991) with a modified *trnT*‐*trnL* forward primer (Cronn et al., 2002).

Amplified product from each region was diluted 200×, then 2 μL was added to 8 μL of Hi‐Di formamide and 0.15 μL of GeneScan 1200 LIZ Size Standard (Applied Biosystems, Foster City, California, USA). Future studies could increase the throughput of FAFLPs by fluorescently labeling the three primer sets and running PCR on a mixed sample, co‐amplifying all three regions. For the current study, we chose to separate species and region to reduce potential error in the creation of the fragment size key. Fragment lengths were resolved using capillary electrophoresis (ABI 3730 DNA Analyzer; Applied Biosystems) and sized to the nearest base pair using GeneMapper 4.0 software (Applied Biosystems). The fragment length was determined by a peak in relative fluorescent units (RFUs).

Visualization with GeneMapper 4.0 showed that many samples contained multiple peaks and peak height varied depending on region amplified, PCR run, and species. When visualization showed multiple peaks, this could be due to multiple binding sites, primer dimers, contamination, or noise. Because of the large variation in peak height, this could not be determined by simple RFU cutoffs and incidences of multiple peaks had to be determined within the context of the amplified region, PCR run, and species. Specifically, in some species, the selected primer sets had multiple binding sites (see Results), which led to two or more tall peaks that were consistent across individuals within a species. In this case, up to four peak heights were recorded. A peak was considered a primer dimer if the length of the fragment was less than 150 bp and was consistently present in multiple samples in the same PCR run. Contamination within a plate was identified when multiple species across distantly related taxa on the same PCR run were resolved as the same size fragment length; these peaks were removed. Finally, a peak was considered noise if there were one or more peaks within the same sample that were at least 10× higher than the peak in question.

Species were considered to have a unique identifier if at least one region differed in length from that of another species by at least one base pair. Fragment lengths or a range of fragment lengths associated with each species were recorded and categorized differently. In the cases where fragment lengths varied within a species by less than 15 bp, this was categorized as interspecific variation. In the cases where fragment lengths were more than 15 bp, this was categorized as a highly variable species because a consistent identifier for that region could not be found. In the cases where only one sample was resolved for a particular region, and thus the length could not be verified by a replicate, a stricter standard was used. Specifically, a sample with a single replicate was recorded only if the species had a clear, high peak height (>2000 RFU) and good ladder size quality (SQ > 0.4, this score reflects how well the data from the GeneScan 1200 LIZ Size Standard [Applied Biosystems] matches expected values), or, if the sample's peak height was lower, it was recorded if the fragment size could be confirmed by a closely related species in the current or other published studies.

### Sanger sequencing congeneric DNA

Because congeners can be difficult to distinguish, we compared FAFLPs and Sanger sequencing in identifying closely related species. The *trnL* intron that was used to establish fragment size profiles was also sequenced for individuals of species within the genera *Alnus* Mill., *Betula* L., *Carex* L., *Cornus* L., *Fragaria* L., *Picea* A. Dietr., *Populus* L., *Ribes* L., and *Rosa* L. (Table 3). The *trnL* intron was selected for comparison because it is amplified with established primer sets (Taberlet et al., 1991), it contains a short and stable secondary structure (i.e., the P6 loop, useful for identifying highly degraded samples [Taberlet et al., 2007]), and of the three regions targeted for the FAFLP analysis, it is the most easily and consistently resolved (Taggart et al., 2011). DNA was extracted using the 2% CTAB method, cleaned using the 5% CTAB method, and amplified using methods described above. Amplified DNA was cleaned using ExoSAP (exonuclease 1 10 units·μL^−1^ [New England Biolabs M0293S; New England Biolabs, Ipswich, Massachusetts, USA] and shrimp alkaline phosphatase 1 unit·μL^−1^ [New England Biolabs M0371S]) following the manufacturer's protocol. Big Dye sequencing reactions and bidirectional sequencing were performed on an ABI 3730 DNA Analyzer (Applied Biosystems) carried out by the University of Alberta Molecular Biology Facility.

**Table 3:** Size key of species and resolved fragment lengths (in base pairs) for three cpDNA regions: the *trnT*‐*trnL* intergenic spacer, the *trnL* intron, and the *trnL*‐*trnF* intergenic spacer.^a^^,^ b

Family	Species^c^	Replicates	*trnT‐trnL*	*trnL*	*trnL‐trnF*
Amaranthaceae	*Blitum capitatum* L. subsp. *capitatum*	2	792–793	x	x
	*Chenopodium album* L.	1	813*/823*	x	x
Apiaceae	*Cicuta maculata* L.	1	846*	559*	x
	*Heracleum maximum* W. Bartram	2	840/430	571	447
	*Hieracium umbellatum* L.	2	x	509	442
	*Osmorhiza depauperata* Phil.	1	839	574	430
	*Sanicula marilandica* L.	2	x	571–572	x
Apocynaceae	*Apocynum androsaemifolium* L.	3	815	418	397
Araliaceae	*Aralia nudicaulis* L.	5	852–853	575	440
Asparagaceae	*Maianthemum canadense* Desf.	3	x	601–602	432
	*Smilacina stellata* (L.) Desf.	3	707*	602	417*
Asteraceae	*Achillea millefolium* L.	5	562	491	425–426
	*Achillea sibirica* Ledeb.	4	768*	491	426–427
	*Artemisia campestris* L.	2	771*	495	440
	*Bidens cernua* L.	1	563*	601/503	396*
	*Cirsium arvense* (L.) Scop.	3	873*	508	v
	*Erigeron philadelphicus* L.	3	x	453*	324
	*Eurybia conspicua* (Lindl.) G. L. Nesom	3	x	495	432*
	*Matricaria discoidea* DC.	2	547*	492–493	440
	*Petasites frigidus* (L.) Fr. var. *palmatus* (Aiton) Cronquist	2	664	511	x
	*Petasites frigidus* var. *sagittatus* (Pursh) Chern.	2	876–881	489	420
	*Solidago canadensis* L.	4	x	500	v
	*Solidago spathulata* DC.	3	x	500	452–458
	*Sonchus arvensis* L. subsp. *uliginosus* (M. Bieb.) Nyman	2	642	507–508	417
	*Symphyotrichum boreale* (Torr. & A. Gray) Á. Löve & D. Löve	1	x	x	432
	*Symphyotrichum ciliolatum* (Lindl.) Á. Löve & D. Löve	5	x	504	432
	*Symphyotrichum laeve* (L.) Á. Löve & D. Löve var. *laeve*	1	896*	504	432
	*Symphyotrichum lanceolatum* (Willd.) G. L. Nesom subsp. *hesperium* (A. Gray) G. L. Nesom	1	x	x	432
	*Tanacetum vulgare* L.	2	642	503	440
	*Taraxacum officinale* F. H. Wigg.	4	621–622	522	402
	*Tripleurospermum inodorum* (L.) Sch. Bip.	2	x	494*	518*
Betulaceae	***Alnus alnobetula*** **(Ehrh.) K. Koch subsp. ** ***crispa*** **(Aiton) Raus**	3	x	602–603	464
	***Alnus incana*** **(L.) Moench subsp. ** ***tenuifolia*** **(Nutt.) Breitung**	4	x	603–605	464
	***Betula glandulosa*** **Michx.**	2	v	440	477
	***Betula occidentalis*** **Hook.**	2	1042–1043/1033	440	446
	***Betula papyrifera*** **Marshall**	4	1043	440	475–476
	***Betula pumila*** **L.**	2	x	440	476*
	*Corylus cornuta* Marshall	4	854	602–603	470
Boraginaceae	*Mertensia paniculata* (Aiton) G. Don	2	780–781	553	453
Brassicaceae	*Arabidopsis lyrata* (L.) O'Kane & Al‐Shehbaz	1	x	576	v
	*Lepidium densiflorum* Schrad.	1	x	590*	x
	*Thlaspi arvense* L.	1	x	401*	x
Campanulaceae	*Campanula rotundifolia* L.	3	831	588–589	x
Caprifoliaceae	*Linnaea borealis* L.	4	804	578–579	447–448
	*Lonicera dioica* L.	2	813/175/210/365	583	443*
	*Lonicera involucrata* (Richardson) Banks ex Spreng.	3	x	587	442–448
	*Symphoricarpos albus* (L.) S. F. Blake	3	815*	587	397*
Caryophyllaceae	*Cerastium nutans* Raf.	1	537*	668*	448*
	*Moehringia lateriflora* (L.) Fenzl	1	707*	629*	417*
	*Stellaria longifolia* Muhl. ex Willd.	4	642/633	637–638	433
Celastraceae	*Parnassia palustris* L.	1	x	686*	382*
Colchicaceae	*Disporum trachycarpum* (S. Watson) Benth. & Hook. f.	4	1008	582	483
Cornaceae	***Cornus canadensis*** **L.**	3	857	582–584	434
	***Cornus stolonifera*** **Michx.**	2	x	590	423*
Cyperaceae	*Carex aenea* Fernald	4	626	689–694	456/444
	***Carex aurea*** **Nutt.**	2	v	686	437
	*Carex bebbii* (L. H. Bailey) Olney ex Fernald	2	624–625	x	x
	*Carex brunnescens* (Pers.) Poir.	1	626*	x	x
	*Carex concinna* R. Br.	1	v	334–337/616*	x
	***Carex crawfordii*** **Fernald**	3	623–625	679	x
	***Carex disperma*** **Dewey**	4	627–628/262/277/618–619	689	443
	*Carex magellanica* Lam. subsp. *irrigua* (Wahlenb.) Hiitonen	1	426/417	x	x
	*Carex utriculata* Boott	2	426/417	x	x
	*Scirpus microcarpus* J. Presl & C. Presl	3	x	690	x
Dryopteridaceae	*Dryopteris carthusiana* (Vill.) H. P. Fuchs	2	x	x	375
Elaeagnaceae	*Shepherdia canadensis* (L.) Nutt.	4	887	550	476
Equisetaceae	*Equisetum arvense* L.	3	x	334	458/431
	*Equisetum hyemale* L.	2	785–799	333	280–281
	*Equisetum palustre* L.	1	x	606*	281
	*Equisetum pratense* Ehrh.	2	x	334	345
	*Equisetum scirpoides* Michx.	3	x	325–333	366
	*Equisetum sylvaticum* L.	4	x	306	345
Ericaceae	*Arctostaphylos uva‐ursi* (L.) Spreng.	5	960/951	575–576	262–263
	*Empetrum nigrum* L.	1	178*	483*	345*
	*Moneses uniflora* (L.) A. Gray	2	x	575	310
	*Orthilia secunda* (L.) House	4	v	593	315
	*Pyrola asarifolia* Michx.	4	920	623*	321
	*Pyrola chlorantha* Sw.	4	917–918	580–581	x
	*Rhododendron groenlandicum* (Oeder) Kron & Judd	2	x	581	452*
	*Vaccinium microcarpum* (Turcz. ex Rupr.) Schmalh.	4	x	563	472–773
	*Vaccinium myrtilloides* Michx.	2	x	561–562	x
	*Vaccinium vitis‐idaea* L.	5	x	567	461
Fabaceae	*Astragalus cicer* L.	1	659*	623	x
	*Lathyrus ochroleucus* Hook.	3	x	510	176
	*Medicago sativa* L.	2	547	x	221*
	*Melilotus albus* Medik.	4	1147–1148/1138	310	205
	*Melilotus officinalis* (L.) Lam.	3	1149	319	216
	*Trifolium hybridum* L.	3	x	615–617	203–209
	*Trifolium pratense* L.	3	x	585	x
	*Trifolium repens* L.	4	x	617/305	203
	*Vicia americana* Muhl. ex Willd.	4	x	522	179
Grossulariaceae	*Ribes americanum* Mill.	3	1122–1123/1112–1113	x	403*
	***Ribes glandulosum*** **Grauer**	3	1103/1193–1194	586	x
	***Ribes hirtellum*** **Michx.**	2	1105/1096	586	x
	*Ribes hudsonianum* Richardson	1	1121*/1112*	586	x
	***Ribes lacustre*** **(Pers.) Poir.**	5	1128/1119	585	411
	***Ribes oxyacanthoides*** **L.**	4	1117–1119/1108–1109/1127	319–320	x
	***Ribes triste*** **Pall.**	5	1109–1110	580	411
Iridaceae	*Sisyrinchium montanum* Greene	4	740–741	551	308
Juncaceae	*Juncus balticus* Willd.	2	811/625	679*	v
Lamiaceae	*Agastache foeniculum* (Pursh) Kuntze	3	602*	v	369–370
	*Galeopsis tetrahit* L.	4	x	v	342
	*Mentha arvensis* L.	1	x	565*	x
	*Scutellaria galericulata* L.	3	740	553–554	386
Lilaceae	*Lilium philadelphicum* L.	2	x	608	255
	*Streptopus amplexifolius* (L.) DC.	2	x	v	454–458
Lycopodiaceae	*Diphasiastrum complanatum* (L.) Holub	4	420/223/411	590	457
	*Lycopodium annotinum* L.	4	439/457	589–590	438
	*Lycopodium obscurum* L.	3	423	598	961
Myricaceae	*Myrica gale* L.	1	x	589*	x
Onagraceae	*Chamaenerion angustifolium* (L.) Scop.	4	x	603–604	504/497
Ophioglossaceae	*Botrypus virginianus* (L.) Michx.	2	x	x	452
Orchidaceae	*Corallorhiza maculata* (Raf.) Raf.	3	x	772/898	471/430*
	*Corallorhiza trifida* Châtel.	2	x	310/542/609	x
	*Galearis rotundifolia* (Banks ex Pursh) R. M. Bateman	1	x	680*	350*
	*Goodyera repens* (L.) R. Br.	1	916*	663	480
	*Platanthera hyperborea* (L.) Lindl.	1	882*	610–620	394*/433*
	*Platanthera obtusata* (Banks ex Pursh) Lindl.	1	906*	619	x
	*Platanthera orbiculata* (Pursh) Lindl.	1	x	600*	492*
Orobanchaceae	*Castilleja miniata* Douglas ex Hook.	4	x	548–553	432–433
	*Melampyrum lineare* Desr.	4	803	544	394
	*Rhinanthus minor* L. subsp. *groenlandicus* (Chabert) Neuman	3	791–792/782–783	x	x
Papaveraceae	*Corydalis aurea* L.	1	x	553*	x
Pinaceae	*Abies balsamea* (L.) Mill.	3	470	554–555	465
	*Larix laricina* (Du Roi) K. Koch	3	472/463	548*	x
	***Picea glauca*** **(Moench) Voss**	6	470/461	559–560	460–465
	***Picea mariana*** **(Mill.) Britton, Sterns & Poggenb.**	5	469/460	559–560	460
	*Pinus banksiana* Lamb.	3	501/492	557	464
Plantaginaceae	*Plantago major* L.	5	764	578	426
	*Veronica americana* (Raf.) Schwein. ex Benth.	1	x	553	405
Poaceae	*Beckmannia syzigachne* (Steud.) Fernald	4	890	609	421
	*Bromus ciliatus* L.	3	v	647	v
	*Bromus inermis* Leyss.	2	v	649	443–444/394
	*Calamagrostis canadensis* (Michx.) P. Beauv.	4	876	490	420
	*Elymus trachycaulus* subsp. *trachycaulus* (Link) Gould ex Shinners	3	668	641–645/423–428	430–432/394
	*Festuca saximontana* Rydb.	2	867–874	571*	v
	*Hordeum jubatum* L.	3	661/652	634	x
	*Koeleria macrantha* (Ledeb.) Schult.	2	842	406	x
	*Leymus innovatus* (Beal) Pilg. subsp. *innovatus*	4	x	557	497
	*Oryzopsis asperifolia* Michx.	4	861–884	600	421–424
	*Phalaris arundinacea* L.	1	880*	v	420–425/349
	*Phleum pratense* L.	5	881–882	608–609	425
	*Piptatheropsis pungens* (Torr. ex Spreng.) Romasch.	4	358–359/349–350	597–600	424–426/394
	*Poa compressa* L.	1	x	611	432
	*Poa palustris* L.	3	882	597	425/394/444
	*Poa pratensis* L.	3	v	620	464*
	*Schizachne purpurascens* (Torr.) Swallen	4	816/740/806	605	417/394
Polemoniaceae	*Collomia linearis* Nutt.	3	x	592–593	440–441
Polygonaceae	*Rumex occidentalis* S. Watson	1	692*	623*	x
Primulaceae	*Lysimachia borealis* (Raf.) U. Manns & Anderb.	5	x	557	336–337
Ranunculaceae	*Actaea rubra* (Aiton) Willd.	5	746–747	542	459
	*Anemone canadensis* L.	2	x	608	448
	*Anemone patens* L.	4	x	571	507
	*Anemone virginiana* L.	1	x	561*	476*
	*Caltha palustris* L.	5	681	564	457
	*Coptidium lapponicum* (L.) Gand. ex Rydb.	1	x	544*	492*
	*Delphinium glaucum* S. Watson	1	760*	v	417–429
	*Ranunculus acris* L.	2	761	647*	444*/349*
	*Ranunculus sceleratus* L.	1	x	565*	x
	*Thalictrum venulosum* Trel.	4	746–748	609–615	469
Rosaceae	*Agrimonia striata* Michx.	1	x	539*	428*
	*Amelanchier alnifolia* (Nutt.) Nutt. ex M. Roem.	5	x	586	484
	***Fragaria vesca*** **L.**	2	998	490	497
	***Fragaria virginiana*** **Mill.**	4	x	490	428–430/394
	*Geum macrophyllum* Willd.	3	x	615	476–477
	*Potentilla norvegica* L.	3	x	599–601	432–492
	*Potentilla palustris* (L.) Scop.	2	918	580	321
	*Prunus pensylvanica* L. f.	1	760*	560*	417*/488*
	*Prunus virginiana* L.	4	920*	592	210/433
	***Rosa acicularis*** **Lindl.**	5	x	616–618	482
	***Rosa woodsii*** **Lindl.**	3	525–526	617	482
	*Rubus arcticus* L.	2	x	569	493
	*Rubus chamaemorus* L.	3	x	556	483
	*Rubus idaeus* L.	5	501*	556	476
	*Rubus pubescens* Raf.	5	501*	569	492
	*Sibbaldia tridentata* (Aiton) Paule & Soják	1	x	499	x
	*Sorbus scopulina* Greene	2	x	586	484*
Rubiaceae	*Galium boreale* L.	2	846	607	483
	*Galium trifidum* L.	2	x	592	442*
	*Galium triflorum* Michx.	2	x	585	470*
Salicaceae	***Populus balsamifera*** **L.**	5	x	653	399–403
	***Populus tremuloides*** **Michx.**	5	525–526	693–695	391–392
	*Salix myrtillifolia* Andersson	1	546	652	432
	*Salix* L. spp.	4	547	653–654	422–432
Santalaceae	*Comandra umbellata* (L.) Nutt.	3	x	572–573	182
	*Geocaulon lividum* (Richardson) Fernald	2	697	578	375
Saxifragaceae	*Mitella nuda* L.	4	377–378	537/580	438*
Typhaceae	*Typha latifolia* L.	3	x	x	389
Urticaceae	*Urtica dioica* L.	3	772	475	443
Violaceae	*Viola adunca* Sm.	5	406–407/397–398	583	443
	*Viola canadensis* L.	5	406/397	583	315
	*Viola palustris* L.	1	405	547	437
	*Viola renifolia* A. Gray	3	377–378	538	438

^a^Plant species were collected from the boreal forest of western Canada.

^b^x = region where amplification failed for a species; v = highly variable species (>15 bp) where a consistent and useful identifier for that region could not be found; * = fragment length that was found in only one replicate and could not be confirmed by a closely related species in the current or other published studies. Ranges are provided when variability was found for a specific fragment length. Lengths from multiple binding sites are separated by a forward slash (/).

^c^The *trnL* intron was sequenced for the species in bold.

Sequence data were manually edited in Geneious version 11.0.5 (Kearse et al., 2012) by replacing bases denoted as “N” that were clearly either G, C, A, or T, based on a distinct single peak. Poor quality 5′ and 3′ ends were trimmed (error probability limit = 0.01) and heterozygotes were detected using the *heterozygotes* plugin (peak similarity = 50%). Bidirectional reverse sequences were aligned using the Geneious de novo assembly alignment tool. Bidirectional sequences were manually searched for inconsistencies, edited if needed (all heterozygotes were edited or replaced by International Union of Pure and Applied Chemistry [IUPAC] ambiguity codes), and the consensus sequence was extracted.

### Comparing identification success between FAFLPs and sequencing

All sequencing data were verified in the National Center for Biotechnology Information (NCBI) GenBank databases using a nucleotide BLAST search. Species were successfully identified when sequences formed monophyletic clades that include one member of the genus and not the others. Clades were built using neighbor‐joining based on the Tamura–Nei genetic distance model (global alignment with free end gaps, cost matrix: 65% similarity) implemented through Geneious version 11.0.5 (Kearse et al., 2012). A Pearson's chi‐squared test was used to evaluate whether sequencing method (FAFLP vs. Sanger) influenced success of species identification.

### Testing the effect of sample richness on species detection success: Simulations and experimental mock communities

To theoretically determine the relationship between species richness of a sample (i.e., a soil core) and species detection success within a defined regional species pool (i.e., our data set of 193 species), we simulated 100 random draws of 10 to 180 species, in increments of 10 species. The number of ambiguities (unresolved species identifications) was determined for each draw for combinations of one, two, or all three cpDNA regions (Appendix S1). In addition to the simulated communities, we also experimentally manipulated richness of samples for species comprising common types of boreal forest. Species lists for two common boreal forest ecosites—those dominated by jack pine (*Pinus banksiana*) (“Pine”) and mixedwood co‐dominated by aspen (*Populus tremuloides*) and white spruce (*Picea glauca*) (“Mixedwood”)—were populated using characteristic species (Beckingham and Archibald, 1996) (Appendix S2). Mock communities consisted of two, four, or eight known species (*n* = 3) randomly drawn from their respective forest ecosite (Appendix S3). The intent of these mock communities was to test detection success within mixed samples consisting of species likely to co‐occur rather than a random assemblage of the entire regional pool.

To determine the number of species identified in each of the tests above, we matched peak profiles from the mixed samples with fragment lengths comprising the reference of 193 species. To create the reference species database, every possible combination of fragment sizes across the three cpDNA regions for each species was created and each served as a unique “code” (Appendix S1). That is, species with multiple binding sites or showing intraspecific variation will have multiple codes encompassing the variation present in fragment sizes. Regions that were not amplified (listed as “x” or “v” in Table 3) were given a value of 0. Regions that were resolved only once (marked with an * in Table 3) were accepted as true values.

## RESULTS

### Species size key of FAFLPs

None of the three regions could be amplified for 16 of the 209 species collected. Of the remaining 193 species, fragment lengths were resolved for 90%, 58%, and 78% of species for the *trnL* intron, the *trnT*‐*trnL* intergenic spacer, and the *trnL*‐*trnF* intergenic spacer, respectively.

Using all three regions, 89 of the remaining 193 species did not produce unique size profiles, i.e., some fragment lengths were identical across multiple species (Table 3). Twenty‐one of these species were closely related (i.e., congeners or confamiliars), and 68 were distantly related species. Unique size profiles were created for 54% of species using FAFLPs of all three regions, and for 65% of genera in this study. Using one region, 17%, 25%, and 16% of species could be distinguished by differences in fragment sizes of the *trnL* intron, the *trnT*‐*trnL* intergenic spacer, and the *trnL*‐*trnF* intergenic spacer, respectively. Using two regions, 30%, 25%, and 39% of species could be distinguished using combinations of the *trnT*‐*trnL* intergenic spacer and *trnL* intron, the *trnT*‐*trnL* intergenic spacer and *trnL*‐*trnF* intergenic spacer, and the *trnL* intron and *trnL*‐*trnF* intergenic spacer, respectively.

The three regions varied in intraspecific variation as well as the occurrence of multiple binding sites. For the *trnT*‐*trnL* intergenic spacer, *trnL* intron, and *trnL*‐*trnF* intergenic spacer, 30, seven, and 15 species had multiple binding sites that caused multiple peak heights. Furthermore, for the *trnT*‐*trnL* intergenic spacer, *trnL* intron, and *trnL*‐*trnF* intergenic spacer, 29, 37, and 28 species showed intraspecific variation (<15 bp). In total, 75 species had some amount of intraspecific variation, 47 species had multiple binding sites, and 17 species had a large range in fragment length (>15 bp, and up to hundreds of base pairs) that was found across individuals in the same species with relatively high confidence (peak height >5000 RFU).

### Comparing identification success between FAFLPs and sequencing

Almost all GenBank searches on sequences produced a closely related species in the expected genera with high query cover (mean = 99%) and pairwise identity (mean = 99%). Of 79 samples sequenced, 76 were of high quality. The exceptions were two *Fragaria vesca* L. sequences that produced a BLAST search result in the *Festuca* L. genus, and one *Picea glauca* sequence that produced a BLAST result of *Pinus* L. These samples were considered contamination, representing 4% of the sequenced samples, and not considered further.

**Figure 1:**
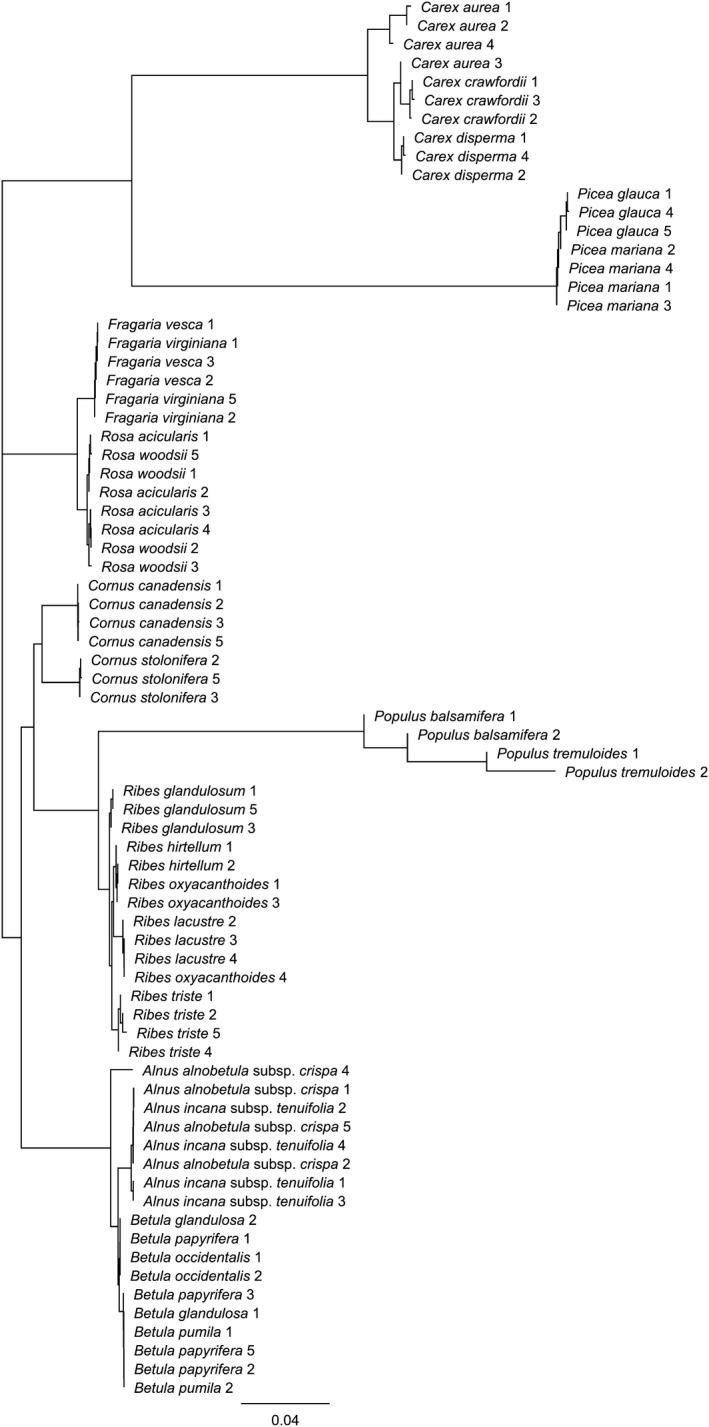
Phylogenetic tree of congeners. Tree was built using neighbor‐joining based on the Tamura–Nei genetic distance model on the *trnL* intron of each individual. Numbers represent an individual of a given species.

Species belonging to eight of the nine genera sequenced were placed in monophyletic clades containing only that genus (Fig. 1), with one exception: an individual *Alnus alnobetula* (Ehrh.) K. Koch subsp. *crispa* (Aiton) Raus was placed just outside of a monophyletic *Alnus* group. No species within *Alnus*,* Betula*,* Rosa*, or *Fragaria* could be distinguished based on sequences of the *trnL* intron. Both members of the *Cornus* genus could be distinguished with sequencing, but only some members of the remaining species formed truly monophyletic clades. Most *Carex* species could be distinguished with the exception of *C. aurea* Nutt., for which one sample was placed slightly outside of a monophyletic group. *Ribes triste* Pall. and *R. glandulosum* Grauer could be distinguished, but *R. hirtellum* Michx. and *R. lacustre* (Pers.) Poir., although in separate clades from each other, were both grouped with *R. oxyacanthoides* L. *Ribes oxyacanthoides* showed a much smaller fragment length for the *trnL* intron than other members of the *Ribes* genus (Table 3), so it is possible that this misplacement is due to *R. oxyacanthoides* missing an important characteristic section of DNA. Finally, *Populus tremuloides* and *Picea glauca* formed monophyletic clades, but their corresponding congeners did not produce true monophyly.

Of the 24 congeners tested, eight (33%) could be distinguished by sequencing of the *trnL* intron, and 10 (42%) could be distinguished by FAFLPs of the *trnL* intron alone (Table 4). Detection success did not significantly differ between FAFLPs and sequencing of the *trnL* intron alone (*χ*²_(1,24)_ = 0.36, *P* = 0.551), but detection success was higher when FAFLPs of all three regions were compared with that of Sanger sequencing of the *trnL* intron (*χ*²_(1,24)_ = 8.39, *P* = 0.004).

**Table 4:** Species comparison of identification success with size fragment *trnL* alone, with a combination of the *trnL* intron, *trnT‐trnL*, and *trnL‐trnF* intergenic spacers, and with Sanger sequencing data of the *trnL* intron

Genus	Species	*n*	Sequencing^a^		FAFLPs^b^				
			Genus	Species	*trnT‐trnL*	*trnL*	*trnL‐trnF*	Unique?	*trnL* alone?
*Alnus*	*alnobetula* subsp. *crispa*	4	N	N	x	602–603	464	N	N
*Alnus*	*incana* subsp. *tenuifolia*	4	N	N	x	603–605	464	N	N
*Betula*	*glandulosa*	2	Y	N	v	440	477	Y	N
*Betula*	*occidentalis*	2	Y	N	1042–1043/1033	440	446	Y	N
*Betula*	*papyrifera*	4	Y	N	1043	440	475–476	N	N
*Betula*	*pumila*	2	Y	N	x	440	476*	N	N
*Carex*	*aurea*	4	Y	N	v	686	437	Y	Y
*Carex*	*crawfordii*	3	Y	Y	623–625	679	x	Y	Y
*Carex*	*disperma*	3	Y	Y	627–628/262/277/618–619	689	443	Y	Y
*Cornus*	*canadensis*	4	Y	Y	857	582–584	434	Y	Y
*Cornus*	*stolonifera*	3	Y	Y	x	590	423*	Y	Y
*Fragaria*	*vesca*	3	Y	N	998	490	497	Y	N
*Fragaria*	*virginiana*	3	Y	N	x	490	428–430/394	Y	N
*Picea*	*glauca*	4	Y	Y	470/461	559–560	460–465	Y	N
*Picea*	*mariana*	4	Y	N	469/460	559–560	460	Y	N
*Populus*	*balsamifera*	2	Y	N	x	653	399–403	Y	Y
*Populus*	*tremuloides*	2	Y	Y	525–526	693–695	391–392	Y	Y
*Ribes*	*glandulosum*	3	Y	Y	1103/1193–1194	586	x	Y	N
*Ribes*	*hirtellum*	2	Y	N	1105/1096	586	x	Y	N
*Ribes*	*lacustre*	3	Y	N	1128/1119	585	411	Y	Y
*Ribes*	*oxyacanthoides*	3	Y	N	1117–1119/1108–1109/1127	319–320	x	Y	Y
*Ribes*	*triste*	4	Y	Y	1109–1110	580	411	Y	Y
*Rosa*	*acicularis*	4	Y	N	x	616–618	482	N	N
*Rosa*	*woodsii*	4	Y	N	525–526	617	482	N	N

Note

FAFLP = fluorescent amplified fragment length polymorphism; *n* = number of samples.

^a^N = sample was not successfully identified to genus and species; Y = sample successfully identified to genus and species.

^b^Fragment sizes of all three cpDNA regions are presented as well as whether a unique size profile was created for a given species. x = region where amplification failed for a species; v = highly variable species (>15 bp) where a consistent and useful identifier for that region could not be found. * = fragment length that was found in only one replicate and could not be confirmed by a closely related species in the current or other published studies. Ranges are provided when variability was found for a specific fragment length. Lengths from multiple binding sites are separated by a forward slash (/).

### The effect of sample richness on species detection success: Simulations and experimental mock communities

**Figure 2:**
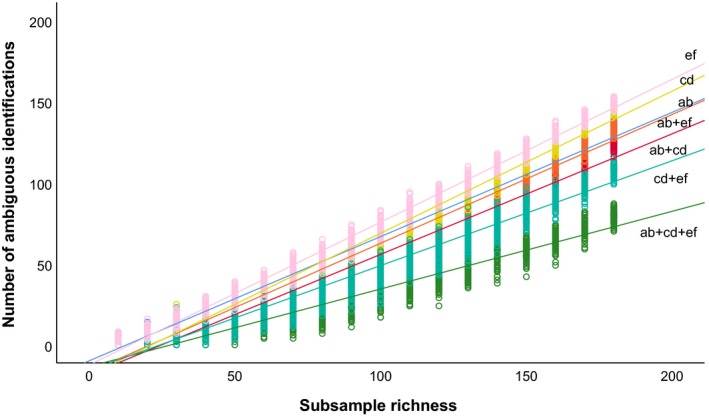
Relationship between correct species identification and species richness of a sample. Lines represent linear regressions for cpDNA regions used in isolation or combination for species identification. Data are simulated from 100 random draws of the regional pool. ab = *trnT‐trnL* intergenic spacer; cd = *trnL* intron; ef = *trnL‐trnF* intergenic spacer.

**Figure 3:**
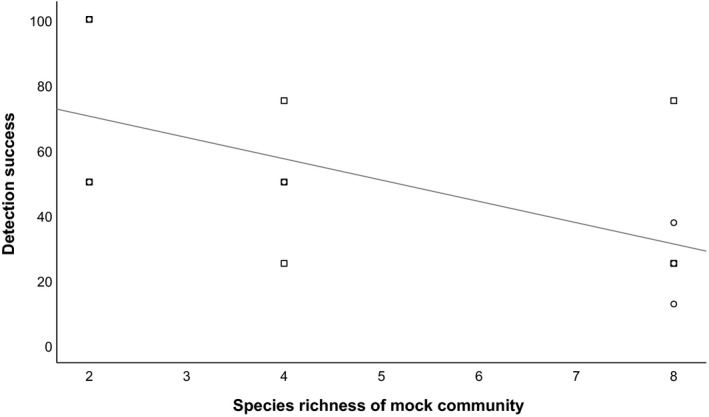
Relationship between detection success of species in experimental mock communities and species richness of that community. Squares represent outcomes from mock communities representing *Pinus banksiana*–dominated communities and circles represent those from mixedwood boreal forests.

In simulated communities, the number of ambiguous species identifications increased with species richness of the artificially subsampled communities, and also depended on the combination of cpDNA used (Fig. 2). Specifically, using any single cpDNA region resulted in higher ambiguous species identifications than a combination of regions. Similar to simulated draws of the regional pool, in experimental mock communities, detection success declined with richness of subsamples (*F*_2,18_ = 4.83, *P* = 0.029) (Fig. 3). Some species, such as *Leymus innovatus* (Beal) Pilg. and *Chamaenerion angustifolium* (L.) Scop., appeared particularly resistant to detection (Appendix S4). Without knowledge of the membership of mock communities, ambiguous species identifications can arise (Appendix S4). Biases in detection based on species identity would not be present in the simulated communities and further reduces the upper limits of detection success.

## DISCUSSION

### Expanding reference FAFLPs for use in identifying roots to species

Our study doubles the number of reference species available for future studies on root identification. To date, this study is the largest plant species pool examined (193 vs. 95 species [Taggart et al., 2011]). We achieved moderate success in identifying unique size profiles for this large pool of plant species occurring in the western Canadian boreal forest; using all three cpDNA regions differentiated 54% of the 193 species for which DNA extractions were successful. Through further testing on simulated communities, our study demonstrates that increases in richness of the regional species pool decreases the probability of finding unique FAFLPs across species. In other words, as reference databases are populated with fragment size profiles of an increasing number of species, redundancies in FAFLPs will occur. This result emphasizes the importance of constraining species pools to which sampled roots are compared.

Several species profiled in the current study have also been examined using FAFLPs in past studies (Appendix S5). Specifically, *Abies balsamea* (L.) Mill., *Betula papyrifera* Marshall, *Picea glauca*,* P. mariana*, and *Populus tremuloides* were examined by Randall et al. (2014). Fragment sizes across the three regions from the current study were similar to those in Randall et al. (2014) for *Picea glauca*,* P. mariana*, and *Populus tremuloides*; the two remaining species differed in fragment sizes for at least one region. The current study and Taggart et al. (2011) had 23 species in common, and for 14 of those, fragment sizes were consistent. *Betula papyrifera* and *Elymus trachycaulus* (Link) Gould ex Shinners were the most inconsistent across studies, with fragment sizes varying across two cpDNA regions (Appendix S5). More testing is required to resolve species for which a single replicate was used in Taggart et al. (2011); however, some of the inconsistencies may be due to intraspecific variation. Taken together, we recommend collecting leaves for fragment size profiling when taking soil cores. Leaves will serve as reference fragment size profiles for identifying roots.

A common finding across studies using FAFLPs is the presence of intraspecific variation in fragment lengths. In the current study, 75 of 193 species showed some amount (<15 bp) of intraspecific variation. Randall et al. (2014) found intraspecific variation in all seven tree species studied, and Ridgway et al. (2003) found intraspecific variation in nine of 10 grassland species. Taggart et al. (2011) recorded only one incidence of intraspecific variation in the 49 species that were replicated (i.e., 2–10 replicates), but this could be a result of limited geographical sampling. In the current study, and in the context of the studies mentioned above, intraspecific variation in fragment sizes may be more common than previously assumed (Ridgway et al., 2003; Taggart et al., 2011; Randall et al., 2014). In the current study, intraspecific variation caused a high level of ambiguous fragment lengths among closely related species, but also among unrelated species. In addition, 17 species had lengths that were highly variable within species (>15 bp). These data were treated as unresolved lengths because it is unclear if they represent biological variation. Many of the ambiguities observed in FAFLP size profiles due to intraspecific variation can be limited by restricting the number of species in an identification key for roots to the number of species identified aboveground through field surveys. Making repeated inventories on the study site and using historical records, if available, would increase the probability of including dormant or seasonal species to the species pool. By restricting the species pool of roots, species with the same size polymorphism are less likely to be encountered in analysis. A way forward in exploring ambiguities in size profiles is to test for a phylogenetic signal in intraspecific variation to predict in which taxa this variation will occur.

Multiple peaks are another consistent finding associated with FAFLPs (Ridgway et al., 2003; Taggart et al., 2011; Randall et al., 2014). At the molecular level, multiple peaks may be a result of nonspecific binding or binding to repeated sequences in the genome; both events can generate fragments of different sizes. Although it is usually clear which peak is signal and which is noise in a single‐species sample, in samples containing multiple species it may be difficult to identify an individual species in the presence of multiple peaks, as peak height may vary depending on species and PCR inhibitors present. To improve the throughput of FAFLPs in the present study, we applied a 1 : 200 dilution to the entire plate of PCR product regardless of the band brightness of gel electrophoresis of the PCR product. In the future, it may be necessary to adjust the dilution based on band brightness to standardize the expected peak heights (RFUs). Next steps to improve FAFLP analysis in multiplexing must include developing standard methods for parsing out signal peaks from noise.

Resolving unique FAFLP size profiles is a task that needs development for application to mixed‐species communities. Ideally, one value (i.e., fragment length) is associated with each region for a given species to create a unique size profile. As we show here, fragments may be absent entirely (amplifications failed) or range in size owing to intraspecific variation and multiple binding sites. Many of the size profiles in this study were ambiguous because of variability that causes a range of values or missing peaks and reduces the potential for unique values. Taggart et al. (2011) offer four analytical approaches to identify unknown species using fragment size profiles. Which of the four approaches is used depends on whether one fragment length from one region is enough for identification (liberal) or if all known fragment lengths must be detected (conservative), and whether or not the user limits the species pool to what was detected aboveground (constrained or unconstrained). However, none of these analytical approaches consider multiple peaks or intraspecific variation. Future analyses of fragment size profiles should use approaches that take into consideration that: (1) not all known fragment lengths may be resolved for each species size profile, (2) species detected aboveground are the most likely to be found belowground (except dead, dormant, or seasonally present species), (3) some size profiles include multiple peaks for one region, and, importantly, (4) intraspecific variation occurs. Moving forward, it may be possible to determine the extent of intraspecific variation and frequency of multiple peaks across species by using in silico methods (e.g., analysis of publicly available sequences from databases such as NCBI's GenBank). Moreover, bioinformatics approaches have also been developed to probabilistically assign taxonomy to include uncertainty owing to incomplete reference databases, mislabeled reference sequences, intraspecific variation, and errors in DNA sequences (Somervuo et al., 2016, 2017; Abarenkov et al., 2018). These analytical tools could be adapted to account for uncertainty in fragment lengths to probabilistically assign identities to species in mixed‐species samples of roots.

### Identification success using FAFLPs compared to sequencing

Our second objective in this study was to test the limits of FAFLPs in species identification. Toward this goal, we compared identification success between Sanger sequencing and FAFLPs of congeners, i.e., species within a genus. FAFLPs of the *trnL* intron were unexpectedly resolved as sequencing of the *trnL* intron. This finding is unexpected because sequencing gives more detailed information, i.e., the sequence of hundreds of base pairs, whereas FAFLPs provide only the region length. One implication of these findings is that FAFLPs can be as effective as sequencing in identifying species. Of note, FAFLPs require less time and are more economical than sequencing. The lack of discrimination within *Betula* and *Rosa* is not surprising considering all members of the species studied within these genera hybridize with at least one other species within each of the genera (Brouillet et al., 2010).

Using FAFLPs of all three regions was much more effective than using the sequence of just one region. One implication of this finding is that it may not be as important which molecular techniques are used, rather the number of regions targeted may be the key step to identify species using DNA‐based methods. With barcoding strategies, it has been suggested that the number of regions is more important than the identity of those regions for correct species identification (Fazekas et al., 2008). In this same line, the Barcode of Life Data System (Ratnasingham and Hebert, 2007) recommends the combination of *rbcL* and *matK* regions to identify plant species. Seberg and Petersen (2009) suggest that it is unlikely that a single barcode will allow us to identify more than 70–75% of known species, although a concatenation of four barcodes allowed them to identify 92% of species within the *Crocus* L. (Iridaceae) genus. Regardless of target region, some researchers have suggested there is an “upper limit” on detecting species using barcodes (Fazekas et al., 2009). Using multiple loci to create a barcode seems to be necessary for in‐depth taxonomy, but this may be impractical for applying barcodes to species identification in mixed‐species samples.

We used similar methods to extract DNA for FAFLP and Sanger sequencing, and these methods require development to increase DNA yields. The recovery rates of size lengths for each region in the current study (*trnT*‐*trnL* intergenic spacer: 58%, *trnL* intron: 90%, and *trnL*‐*trnF* intergenic spacer: 78%) are similar to those found in Taggart et al. (2011) (58%, 100%, and 98%, respectively). The higher recovery rates found for the *trnL* intron and the *trnL*‐*trnF* intergenic spacer by Taggart et al. (2011) could be attributed to the use of a different DNA extraction method (DNeasy PowerPlant Pro Kit; QIAGEN) or the different species present in grasslands versus the boreal forest. Additionally, we used a different forward primer for the *trnL*‐*trnF* intergenic spacer, which was more successful in the number of samples amplified (data not shown). This change in forward primer may explain why recovery rate was the same for the *trnT*‐*trnL* intergenic spacer, but lower for the other regions. These results, together with those by Taggart et al. (2011), indicate that the amplification of DNA, especially for plants, may depend on DNA extraction method and PCR inhibitors specific to species (Mommer et al., 2011). Therefore, optimization of extraction and PCR condition based on species may be a prerequisite to using PCR‐based methods in species‐rich systems. Commercial DNA extraction kits such as DNeasy PowerPlant or PowerSoil kits (QIAGEN) are designed to remove impurities such as polysaccharides in leaves or humic acids in soil, but neither kit is optimized for roots. Using both kits (the first optimized for plants and the second optimized for soil) would be expensive, and more importantly, would greatly reduce the concentration of DNA extracted. Although these kits are currently the best option for extracting good quality DNA from roots, it may be possible to adapt the CTAB method to improve yield. There are a variety of “homebrew” extraction methods designed for roots (Linder et al., 2000; Brunner et al., 2001; Khan et al., 2007), but they are often optimized for a limited number of species. In our study, DNA recovery from the second extraction method (Griffiths et al., 2001) was much higher than the first (Roe et al., 2010), even though these samples were nonrandomly chosen from a group that was unsuccessful using the 2% CTAB method. This finding suggests that more troubleshooting and optimization may produce significant advances in DNA extraction purity, especially in combination with commercial DNA extraction kits.

### The importance of species richness and identity on species detection

Low species detection with increased community diversity may be an inevitable outcome across a range of plant identification methods. A corollary of increased diversity is that the abundance of species in the community must decrease if plant density remains intact. Previous studies based on plant surveys have repeatedly found that detection increases with species abundance (Chen et al., 2009; Dennett et al., 2018). Similar to outcomes of plant surveys, we found a negative relationship between species richness (of DNA template) and detection success. Artificially drawing communities from a regional pool across a range of species richness clearly demonstrated that, when a sample contains many species, it is likely their identity will be ambiguous using FAFLPs. That is, the higher the richness, the more likely species will overlap in cpDNA fragment sizes. Our simulations show that the rate of ambiguous identities should decrease when the richness of a sample is low. On one hand, we expect species richness of roots cored from soils to be low, i.e., under 10 (Frank et al., 2010). Our experiments with mock communities, however, demonstrate that even in species‐poor communities (e.g., four species), the average detection success was 50%. This outcome is likely a result of species bias at the DNA extraction and amplification stages. Previous research shows species vary in DNA yields and amplicon yield is positively related to DNA concentration (Karst et al., 2015). Another known bias during amplification is based on fragment size; DNA from species that have smaller cpDNA fragments is more likely to amplify than those with longer fragments (Karst et al., 2015). Detection success in our study was lower than that reported in Taggart et al. (2011), likely because they created mock communities with extracted DNA and, as such, their experiments would be free from extraction biases present among species. One way to circumvent these types of false negatives is to use an aboveground species survey to not only help constrain the expected species pool within a core, but also scrutinize possible false negatives (and false positives).

In conclusion, we recommend that (1) FAFLPs are best suited to confirm rather than discover species from soil cores and (2) FAFLPs are best suited for conditions of low plant diversity and where the sampled species pools are constrained by species known to occur aboveground. In future studies, we suggest using publicly available sequences to understand how intraspecific variation and incidences of multiple peaks function in relation to species, and how we can incorporate these features into analysis of FAFLPs. In addition to expanding analytical methods to resolve species identification, we also suggest development of extraction methods for use on a wide range of plant taxa.

## AUTHOR CONTRIBUTIONS

P.M. and J.K. conceived of the study. P.M. performed field and lab work, data analysis, and wrote the initial draft of the manuscript. M.L. designed the concept for mock communities and performed the lab work and subsequent analysis. All authors revised and approved the final manuscript.

## DATA ACCESSIBILITY

The data that support the findings of this study are openly available in University of Alberta Libraries (UAL) Dataverse (https://doi.org/10.7939/DVN/ZZKSVL).

## Supporting information


**APPENDIX S1.** Generating mock communities for fluorescent amplified fragment length polymorphism analysis.

**APPENDIX S2.** Common species present in two common ecosites of boreal forest in northern Alberta, Canada. These species formed two pools from which mock communities were subsampled.

**APPENDIX S3.** Subsampled communities comprising mock communities designed to test influence of species richness on detection success using fluorescent amplified fragment length polymorphisms.

**APPENDIX S4.** Species resolved in mock communities of “Pine” and “Mixedwood” forest ecosites.

**APPENDIX S5.** Comparison of fragment lengths (number of base pairs) for three cpDNA regions (the *trnT‐trnL* intergenic spacer, the *trnL* intron, and the *trnL‐trnF* intergenic spacer) across studies: (A) the current study compared to Randall et al. (2014), and (B) the current study compared to Taggart et al. (2011).
