# Supplementary material for: Expanding and testing fluorescent amplified fragment length polymorphisms for identifying roots of boreal forest plant species
Source: Appl Plant Sci. 2019 Apr 8;7(4):e01236. doi: 10.1002/aps3.1236 (PMC6476169; doi:10.1002/aps3.1236)
Supplement: Supplementary file 1 — APPENDIX S1. Generating mock communities for fluorescent amplified fragment length polymorphism analysis. [file APS3-7-e01236-s001.docx]

**APPENDIX S1.** Generating mock communities for fluorescent amplified fragment length polymorphism analysis.

***Methods for simulating sampling from regional pool of species***

The document “limit” contains all possible reference values for each region as described below in “Methods for experimentally manipulating mock communities.” In addition, because a fragment length profile such as ‘400 600 x’ is considered ambiguous with ‘400 x x’ (see Methods), ‘x’ was included as a possible value when creating all possible fragment length profiles (i.e., permutations of each fragment profile were made that include all three observed values “nnn”, as well as one or two of the observed values “nxx”, “xnx”, “xxn”, “nnx”, “nxn”, and “xnn”).

library(readr)

limit <- read_csv("C:/Users/Paul Metzler/Desktop/faflp_limitations_R.csv")

Columns that included every combination of regions were created.

limit$TLL <- paste(limit$trnTtrnL,limit$trnL)

limit$TLLF <- paste(limit$trnTtrnL,limit$trnLtrnF)

limit$LLF <- paste(limit$trnL,limit$trnLtrnF)

limit$id <- paste(limit$trnTtrnL,limit$trnL,limit$trnLtrnF)

A subset of the limit data that did not include ‘x’ as a value (the “nnn” permutation) was made for comparison. For example, the “nnx” permutation of ‘400 600 500’ and ‘400 600 100’, if compared to each other, would read as ambiguous, when in fact, they are unique.

nnn <- subset(limit, limit$perm == "nnn", select = c(sp,trnTtrnL,trnL,trnLtrnF,TLL,TLLF,LLF,id))

A function was then made that relies on the above code. The input of the function is the richness of the artificially sampled species from the regional pool. This function is for all cpDNA regions together, but the function was edited each time for each combination of regions.

myFunAll <- function(numSp, ...){

randoSpecies <- sample(unique(limit$sp), numSp)

randoId <- subset(nnn, nnn$sp %in% randoSpecies,

select = c(sp,id))

randoId2 <- subset(limit, limit$sp %in% randoSpecies,

select = c(sp,id))

simple <- function(x,...){

unique(subset(randoId2, randoId2$id %in% x, select = c(sp)))

}

randoId$new <- lapply(randoId$id, simple)

spLength <- function(x,...){

length(unique(subset(randoId2, randoId2$id == x, select = c(sp)))$sp)

}

randoId$length <- lapply(randoId$id, spLength)

library(reshape2)

combo <- melt(subset(randoId, randoId$length > 1,

select = c(sp,id,new,length))$new)

length(unique(combo$sp))

}

myFunAll(100)

### Outputs the number of species that are ambiguous based on

###### the random selection of the input number of species

# resampling using 100 replicates and

## species pools of different sizes (multiples of ten), up to 180

tenSp <- replicate(100, myFunLLF(10))

twentSp <- replicate(100, myFunLLF(20))

thirtSp <- replicate(100, myFunLLF(30))

fortSp <- replicate(100, myFunLLF(40))

fiftSp <- replicate(100, myFunLLF(50))

sixtSp <- replicate(100, myFunLLF(60))

seventSp <- replicate(100, myFunLLF(70))

eightySp <- replicate(100, myFunLLF(80))

ninetySp <- replicate(100, myFunLLF(90))

hundoSp <- replicate(100, myFunLLF(100))

hunTen <- replicate(100, myFunLLF(110))

hunTwen <- replicate(100, myFunLLF(120))

hunThir <- replicate(100, myFunLLF(130))

hunFor <- replicate(100, myFunLLF(140))

hunFift <- replicate(100, myFunLLF(150))

hunSixt <- replicate(100, myFunLLF(160))

hunSevent <- replicate(100, myFunLLF(170))

hunEight <- replicate(100, myFunLLF(180))

# make them into matrices

tenSp <- cbind(tenSp, 10, "lf")

twentSp <- cbind(twentSp, 20, "lf")

thirtSp <- cbind(thirtSp, 30, "lf")

fortSp <- cbind(fortSp, 40, "lf")

fiftSp <- cbind(fiftSp, 50, "lf")

sixtSp <- cbind(sixtSp, 60, "lf")

seventSp <- cbind(seventSp, 70, "lf")

eightySp <- cbind(eightySp, 80, "lf")

ninetySp <- cbind(ninetySp, 90, "lf")

hundoSp <- cbind(hundoSp, 100, "lf")

hunTen <- cbind(hunTen, 110, "lf")

hunTwen <- cbind(hunTwen, 120, "lf")

hunThir <- cbind(hunThir, 130, "lf")

hunFor <- cbind(hunFor, 140, "lf")

hunFift <- cbind(hunFift, 150, "lf")

hunSixt <- cbind(hunSixt, 160, "lf")

hunSevent <- cbind(hunSevent, 170, "lf")

hunEight <- cbind(hunEight, 180, "lf")

# make dataframe

RegTLLF <- rbind(tenSp,twentSp,thirtSp,fortSp,fiftSp,sixtSp,seventSp,eightySp,

ninetySp,hundoSp,hunTen,hunTwen,hunThir,hunFor,hunFift,hunSixt,

hunSevent,hunEight)

RegTLLF <- as.data.frame(RegLLF)

fwrite(RegLLF, file = "RegLLF.csv")

View(RegLLF)

***Methods for experimentally manipulating mock communities***

Our goal was to evaluate detection success of known species added to mock communities. We first created two “communities” populated by characteristic species known to occur in two common boreal forest ecosites; those dominated by jack pine (*Pinus banksiana*) (“Pine”) and mixedwood co-dominated by aspen (*Populus tremuloides*) and white spruce (*Picea glauca*) (“Mixedwood”) (Beckingham and Archibald, 1996) (Appendix S2). We then manipulated richness of samples drawn from each of the respective forest ecosites. Samples consisted of two, four, or eight known species (*n* = 3) (Appendix S3). From each forest ecosite, species were randomly drawn to create the mock communities for DNA extraction.

For DNA extractions, plant tissue was subsampled from original dried and pulverized collections (see Methods). Subsamples were collected with sterilized equipment cleaned between each species and weighed on an analytical scale. The samples contained between 30 and 60 mg of plant tissue as follows:

- 2 species samples ~15 mg⋅species^-1^ for a total of ~30 mg⋅sample^-1^
- 4 species samples ~10 mg⋅species^-1^ for a total of ~40 mg⋅sample^-1^
- 8 species samples ~6 mg⋅species^-1^ for a total of ~50–60 mg⋅sample^-1^

DNeasy PowerPlant Pro Kits (QIAGEN, Hilden, Germany) were used to extract total genomic DNA following manufacturer’s protocols. Amplification and sizing of fragments was performed as described in the Methods section in the article.

To create a reference to which subsequent fragment size profiles would be compared, we listed all combinations of fragment sizes representing each cpDNA region for a single species, i.e., an extended reference table. Regions that were not amplified, listed as ‘x’ or ‘v’ in Table 3, were given a value of 0. Regions resolved only once, i.e., those marked with an * in Table 3, were accepted as true values. For example:

|  | **cpDNA region** | | |
| --- | --- | --- | --- |
| **Species** | ***trnT-trnL*** | ***trnL*** | ***trnL-trnF*** |
| *Arctostaphylos uva-ursi* | 960/951 | 575–576 | 262–263 |

Becomes:

|  | **cpDNA region** | | |  |
| --- | --- | --- | --- | --- |
| **Species** | ***trnT-trnL*** | ***trnL*** | ***trnL-trnF*** | **Unique identifier** |
| *Arctostaphylos uva-ursi* | 960 | 575 | 262 | 960 575 262 |
| *Arctostaphylos uva-ursi* | 960 | 575 | 263 | 960 575 263 |
| *Arctostaphylos uva-ursi* | 960 | 576 | 262 | 960 576 262 |
| *Arctostaphylos uva-ursi* | 960 | 576 | 263 | 960 576 263 |
| *Arctostaphylos uva-ursi* | 951 | 575 | 262 | 951 575 262 |
| *Arctostaphylos uva-ursi* | 951 | 575 | 263 | 951 575 263 |
| *Arctostaphylos uva-ursi* | 951 | 576 | 262 | 951 576 262 |
| *Arctostaphylos uva-ursi* | 951 | 576 | 263 | 951 576 263 |

To compare fragment lengths to those populating the extended reference table, lengths were then gathered into a long format in R using both *Dplyr* and *TidyR* packages (R Core Team, 2018; Wickham and Henry, 2018; Wickham et al., 2018) by sample and cpDNA region, i.e., “marker.” Lengths were rounded both up and down with both values included in the end table.

dna2 <- dna %>%

select(Sample:Size.10) %>%

gather(Size.1:Size.10,

key = size,

value = length) ## gathers fragment lengths, ignoring peak height into 1 column

dna3 <- dna2 %>%

mutate(length=ifelse(is.na(length),000,length)) %>% ## changes NAs to 000

mutate(down = floor(length), up = ceiling(length)) %>% ## Rounding

gather(down:up, key = rnd, value = length2) %>% ## gathers rounded values to 1 column

spread(key = Marker, value = length2) %>%

gather(A_B:E_F, key = marker, value = length) %>%

mutate(length=ifelse(is.na(length),000,length)) %>%

group_by(Sample) %>%

spread(key = marker, value = length) %>%

ungroup()

Filtering by sample, the expand.grid() function was used to create a table with every possible combination of each of the three regions and then they were pasted together to create each possible “code”:

b22 <- dna3 %>%

filter(Sample == "B22") %>% ## Selects all values from one sample

select(A_B:E_F) %>% ## Takes only the columns of fragment lengths

expand.grid() %>% ## Creates all combinations possible

mutate(code= paste(A_B, C_D, E_F)) ## Pastes to singles column called “code”

This table was then semi_joined to the reference table by “code” displaying only the records in the reference table that matched a code in the sample table.

b22spp <- semi_join(spplist, b22)

**Literature Cited**

Beckingham, J. D., and J. H. Archibald. 1996. Field guide to ecosites of Northern Alberta. Special Report 5. Natural Resources Canada, Canadian Forest Service, Northern Forestry Centre, Edmonton, Alberta.

R Core Team. 2018. R: A language and environment for statistical computing. R Foundation for Statistical Computing, Vienna, Austria. Website <https://www.R-project.org/> [accessed 20 November 2018].

Wickham, H., and L. Henry. 2018. *tidyr*: Easily tidy data with 'spread()' and 'gather()' functions. R package version 0.8.0. Website <https://CRAN.R-project.org/package=tidyr> [accessed 20 November 2018].

Wickham, H., R. François, L. Henry, and K. Müller. 2018. *dplyr*: A grammar of data manipulation R package version 0.7.8. Website <https://CRAN.R-project.org/package=dplyr> [accessed 20 November 2018].
