# Supplementary material for: Expanding and testing fluorescent amplified fragment length polymorphisms for identifying roots of boreal forest plant species
Source: Appl Plant Sci. 2019 Apr 8;7(4):e01236. doi: 10.1002/aps3.1236 (PMC6476169; doi:10.1002/aps3.1236)
Supplement: Supplementary file 2 — APPENDIX S2. Common species present in two common ecosites of boreal forest in northern Alberta, Canada. These species formed two pools from which mock communities were subsampled. [file APS3-7-e01236-s002.docx]

**APPENDIX S2.** Common species present in two common ecosites of boreal forest in northern Alberta, Canada. These species formed two pools from which mock communities were subsampled.

| **Forest type** | **Species** | | | |
| --- | --- | --- | --- | --- |
|  | **Tree** | **Shrub** | **Forb** | **Grass** |
| Pine | *Pinus banksiana*  *Populus tremuloides*  *Betula papyrifera*  *Picea glauca*  *Picea mariana* | *Rhododendron*  *groenladicum*  *Alnus crispa*  *Vaccinium vitis-idaea*  *Vaccinium myrtilloides*  *Arctostaphylos uva-ursi*  *Shepherdia canadensis*  *Linnaea borealis*  *Rosa acicularis*  *Amelanchier alnifolia* | *Cornus canadensis*  *Chamaenarion*  *angustifolium*  *Lathyrus ochroleucus* | *Leymus innovatus* |
| Mixedwood | *Populus tremuloides*  *Betula papyrifera*  *Picea glauca*  *Picea mariana*  *Abies balsamea*  *Populus balsamifera* | *Corylus cornuta*  *Alnus crispa*  *Viburnum edule*  *Shepherdia canadensis*  *Salix* spp.  *Linnaea borealis*  *Rosa acicularis*  *Amelanchier alnifolia*  *Prunus* spp. | *Aralia nudicaulis*  *Cornus canadensis*  *Chamaenarion*  *angustifolium*  *Mitella nuda*  *Rubus pubescens*  *Fragaria virginiana*  *Pyrola asarifolia*  *Petasites palmatus* | *Calamagrostis canadensis*  *Leymus innovatus* |
