# Supplementary material for: Expanding and testing fluorescent amplified fragment length polymorphisms for identifying roots of boreal forest plant species
Source: Appl Plant Sci. 2019 Apr 8;7(4):e01236. doi: 10.1002/aps3.1236 (PMC6476169; doi:10.1002/aps3.1236)
Supplement: Supplementary file 3 — APPENDIX S3. Subsampled communities comprising mock communities designed to test influence of species richness on detection success using fluorescent amplified fragment length polymorphisms. [file APS3-7-e01236-s003.docx]

**APPENDIX S3.** Subsampled communities comprising mock communities designed to test influence of species richness on detection success using fluorescent amplified fragment length polymorphisms.

|  |  | **List of species randomly drawn from each forest ecosite** | |
| --- | --- | --- | --- |
| **Species richness of mock community** | **Replicate number** | **Pine** | **Mixedwood** |
| 2 | 1 | *Leymus innovatus* | *Mitella nuda* |
|  |  | *Pinus banksiana* | *Populus tremuloides* |
| 2 | 2 | *Picea glauca* | *Picea mariana* |
|  |  | *Populus tremuloides* | *Picea glauca* |
| 2 | 3 | *Rosa acicularis* | *Linnaea borealis* |
|  |  | *Chamaenarion angustifolium* | *Aralia nudicaulis* |
| 4 | 1 | *Chamaenarion angustifolium* | *Alnus crispa* |
|  |  | *Rhododendron groenlandicum* | *Populus tremuloides* |
|  |  | *Alnus crispa* | *Betula papyrifera* |
|  |  | *Betula papyrifera* | *Linnaea borealis* |
| 4 | 2 | *Pinus banksiana* | *Petasites palmatus* |
|  |  | *Vaccinium vitis-idaea* | *Abies balsamea* |
|  |  | *Lathyrus ochroleucus* | *Leymus innovatus* |
|  |  | *Leymus innovatus* | *Cornus canadensis* |
| 4 | 3 | *Rosa acicularis* | *Picea glauca* |
|  |  | *Picea glauca* | *Picea mariana* |
|  |  | *Chamaenarion angustifolium* | *Cornus canadensis* |
|  |  | *Shepherdia canadensis* | *Viburnum edule* |
| 8 | 1 | *Amelanchier alnifolia* | *Amelanchier alnifolia* |
|  |  | *Linnaea borealis* | *Betula papyrifera* |
|  |  | *Rosa acicularis* | *Mitella nuda* |
|  |  | *Picea mariana* | *Rubus pubescens* |
|  |  | *Arctostaphylos uva-ursi* | *Chamaenarion angustifolium* |
|  |  | *Betula papyrifera* | *Populus tremuloides* |
|  |  | *Chamaenarion angustifolium* | *Corylus cornuta* |
|  |  | *Alnus crispa* | *Salix* spp. |
| 8 | 2 | *Pinus banksiana* | *Picea mariana* |
|  |  | *Rhododendron groenlandicum* | *Amelanchier alnifolia* |
|  |  | *Rosa acicularis* | *Viburnum edule* |
|  |  | *Shepherdia canadensis* | *Alnus crispa* |
|  |  | *Picea mariana* | *Populus tremuloides* |
|  |  | *Cornus canadensis* | *Salix* spp. |
|  |  | *Vaccinium vitis-idaea* | *Cornus canadensis* |
|  |  | *Picea glauca* | *Rubus pubescens* |
| 8 | 3 | *Populus tremuloides* | *Leymus innovatus* |
|  |  | *Pinus banksiana* | *Prunus* spp. |
|  |  | *Cornus canadensis* | *Alnus crispa* |
|  |  | *Alnus crispa* | *Rubus pubescens* |
|  |  | *Vaccinium myrtilloides* | *Picea glauca* |
|  |  | *Picea mariana* | *Rosa acicularis* |
|  |  | *Lathyrus ochroleucus* | *Abies balsamea* |
|  |  | *Rosa acicularis* | *Corylus cornuta* |
