# Supplementary material for: Expanding and testing fluorescent amplified fragment length polymorphisms for identifying roots of boreal forest plant species
Source: Appl Plant Sci. 2019 Apr 8;7(4):e01236. doi: 10.1002/aps3.1236 (PMC6476169; doi:10.1002/aps3.1236)
Supplement: Supplementary file 4 — APPENDIX S4. Species resolved in mock communities of “Pine” and “Mixedwood” forest ecosites. [file APS3-7-e01236-s004.docx]

**APPENDIX S4.** Species resolved in mock communities of “Pine” and “Mixedwood” forest ecosites.

| **Composition of mock community** | **Species resolved?** | **Alternative species identities** |
| --- | --- | --- |
| **Pine** |  |  |
| *Leymus innovatus* | No |  |
| *Pinus banksiana* | Yes |  |
| *Picea glauca* | Yes | *Picea mariana* |
| *Populus tremuloides* | Yes |  |
| *Rosa acicularis* | Yes | *Carex concinna* |
| *Chamaenarion angustifolium* | No |  |
| *Chamaenarion angustifolium* | No | *Botrypus virginianus* |
| *Rhododendron groenlandicum* | Yes |  |
| *Alnus crispa* | No |  |
| *Betula papyrifera* | Yes |  |
| *Pinus banksiana* | No |  |
| *Vaccinium vitis-idaea* | No |  |
| *Lathyrus ochroleucus* | Yes |  |
| *Leymus innovatus* | No |  |
| *Rosa acicularis* | Yes | *Carex concinna* |
| *Picea glauca* | Yes | *Alnus* spp. |
| *Chamaenarion angustifolium* | No |  |
| *Shepherdia canadensis* | Yes |  |
| *Amelanchier alnifolia* | Yes | *Carex concinna* |
| *Linnaea borealis* | No | *Lonicera involucrata* |
| *Rosa acicularis* | No |  |
| *Picea mariana* | No |  |
| *Arctostaphylos uva-ursi* | No |  |
| *Betula papyrifera* | Yes |  |
| *Chamaenarion angustifolium* | No |  |
| *Alnus crispa* | No |  |
| *Pinus banksiana* | No | *Castilleja miniate* |
| *Rhododendron groenlandicum* | Yes |  |
| *Rosa acicularis* | Yes |  |
| *Shepherdia canadensis* | Yes |  |
| *Picea mariana* | Yes |  |
| *Cornus canadensis* | Yes |  |
| *Vaccinium vitis-idaea* | No |  |
| *Picea glauca* | Yes |  |
| *Populus tremuloides* | No |  |
| *Pinus banksiana* | No |  |
| *Cornus canadensis* | Yes |  |
| *Alnus crispa* | No |  |
| *Vaccinium myrtilloides* | No |  |
| *Picea mariana* | No |  |
| *Lathyrus ochroleucus* | Yes |  |
| *Rosa acicularis* | No |  |
| **Mixedwood** |  |  |
| *Mitella nuda* | Yes | *Viola renifolia* |
| *Populus tremuloides* | No |  |
| *Picea mariana* | Yes | *Galeopsis tetrahit* |
| *Picea glauca* | Yes |  |
| *Linnaea borealis* | Yes | *Carex concinna* |
| *Aralia nudicaulis* | Yes | *Streptopus amplexifolius* |
|  |  | *Arabis* spp. |
| *Alnus crispa* | No |  |
| *Populus tremuloides* | No |  |
| *Betula papyrifera* | Yes |  |
| *Linnaea borealis* | Yes |  |
| *Petasites palmatus* | No |  |
| *Abies balsamea* | Yes |  |
| *Leymus innovatus* | No |  |
| *Cornus canadensis* | Yes |  |
| *Picea glauca* | Yes | *Ribes triste* |
| *Picea mariana* | No |  |
| *Cornus canadensis* | Yes |  |
| *Viburnum edule* | No |  |
| *Amelanchier alnifolia* | Yes | *Maianthenum canadensis* |
| *Betula papyrifera* | No | *Symphyotrichum boreale* |
| *Mitella nuda* | No | *Symphyotrichum lanceolatum* |
| *Rubus pubescens* | No | *Sorbus scopulina* |
| *Chamaenarion angustifolium* | No | *Typha latifolia* |
| *Populus tremuloides* | No |  |
| *Corylus cornuta* | No |  |
| *Salix* spp. | Yes |  |
| *Picea mariana* | No | *Sorbus scopulina* |
| *Amelanchier alnifolia* | Yes | *Symphyotrichum boreale* |
| *Viburnum edule* | No | *Symphyotrichum lanceolatum* |
| *Alnus crispa* | No |  |
| *Populus tremuloides* | No |  |
| *Salix* spp. | No |  |
| *Cornus canadensis* | No |  |
| *Rubus pubescens* | No |  |
| *Leymus innovatus* | No |  |
| *Prunus* spp. | No |  |
| *Alnus crispa* | No |  |
| *Rubus pubescens* | Yes |  |
| *Picea glauca* | Yes |  |
| *Rosa acicularis* | No |  |
| *Abies balsamea* | Yes |  |
| *Corylus cornuta* | No |  |
