# Supplementary material for: Expanding and testing fluorescent amplified fragment length polymorphisms for identifying roots of boreal forest plant species
Source: Appl Plant Sci. 2019 Apr 8;7(4):e01236. doi: 10.1002/aps3.1236 (PMC6476169; doi:10.1002/aps3.1236)
Supplement: Supplementary file 5 — APPENDIX S5. Comparison of fragment lengths (number of base pairs) for three cpDNA regions (the trnT‐trnL intergenic spacer, the trnL intron, and the trnL‐trnF intergenic spacer) across studies: (A) the current study compared to Randall et al. (2014), and (B) the current study compared to Taggart et al. (2011). [file APS3-7-e01236-s005.docx]

**APPENDIX S5.** Comparison of fragment lengths (number of base pairs) for three cpDNA regions (the *trnT-trnL* intergenic spacer, the *trnL* intron, and the *trnL-trnF* intergenic spacer) across studies: (A) the current study compared to Randall et al. (2014), and (B) the current study compared to Taggart et al. (2011).

**A**

|  | **Metzler et al.^b,c^** | | | **Randall et al. (2014)^b^** | | |
| --- | --- | --- | --- | --- | --- | --- |
| **Species^a^** | ***trnT-trnL*** | ***trnL* intron** | ***trnL-trnF*** | ***trnT-trnL*** | ***trnL* intron** | ***trnL-trnF*** |
| *Abies balsamea* (3) | 470 | 554–555 | 465 | 475 | 554 | 494/508 |
| *Picea glauca* (6) | 470/461 | 559–560 | 460–465 | 474 | 558–559 | 460/462 |
| *Picea mariana* (5) | 469/460 | 559–560 | 460 | 473–474 | 558–559 | 460/462 |
| *Betula papyrifera* (4) | 1043 | 440 | 475–476 | 1031–1031 | 439 | 460 |
| *Populus tremuloides* (5) | 525-526 | 693–695 | 391–392 | 528/530 | 673/692/694 | 392 |

^a^Numbers in parentheses following species names are replicates for Metzler et al. (i.e., the current study). Replicates vary from one to six in Randall et al. (2014).

^b^Ranges are provided when variability was found for a specific fragment length. Lengths from multiple binding sites are separated by a forward slash (/).

^c^Cells colored green in Metzler et al. indicate values that are within 1 bp of those in Randall et al. (2014); red cells indicate values that are greater than 1 bp. Note that the *trnT-trnL* intergenic spacer was amplified using a modified primer in the current study and, consequently, will be 5 bp shorter than fragments amplified using Taberlet et al. (1991) primers A and B (see Table 2).

**B**

|  | **Metzler et al.^b,c^** | | | **Taggart et al. (2011)^b^** | | |
| --- | --- | --- | --- | --- | --- | --- |
| **Species^a^** | ***trnT-trnL*** | ***trnL* intron** | ***trnL-trnF*** | ***trnT-trnL*** | ***trnL* intron** | ***trnL-trnF*** |
| *Achillea millefolium* (5) | 562 | 491 | 425–426 | 567 | 491/262 | 426 |
| *Amelanchier alnifolia* (5) | x | 586 | 484 | x | 586 | 484 |
| *Artemisia campestris* (1) | 771* | 495 | 440 | x | 495 | 440 |
| *Chenopodium album* (1) | 813*/823* | x | x | 836 | 589 | 416 |
| *Cirsium arvense* (3) | 873* | 508 | v | x | 590 | 456 |
| *Comandra umbellate* (3) | x | 572–573 | 182 | 657 | 573 | 183 |
| *Elymus trachycaulus* (3) | 668 | 641–645/423–428 | 430–432/349 | 675 | 649/515 | 394/432 |
| *Fragaria virginiana* (4) | x | 490 | 428–430/394 | x | 490 | 497 |
| *Galium boreale* (2) | 846 | 607 | 483 | x | 607 | 483 |
| *Hordeum jubatum* (3) | 661/652 | 634 | x | 666 | 635 | 394/406 |
| *Koeleria macrantha* (2) | 842 | 406 | x | 848 | 407 | 418 |
| *Lathyrus ochroleucus* (3) | x | 510 | 176 | x | 511 | 176 |
| *Lepidium densiflorum* (1) | x | 590* | x | 885 | 590 | 596 |
| *Lilium philadelphicum* (2) | x | 608 | 255 | 815 | 608 | 255 |
| *Melilotus officinalis* (3) | 1149 | 319 | 216 | x | 320 | 217 |
| *Poa palustris* (3) | 882 | 597 | 425/394/444 | 887 | 620 | 444 |
| *Potentilla norvegica* (3) | x | 599–601 | v | 823 | 633 | 447 |
| *Sisyrinchium montanum* (4) | 740–741 | 551 | 308 | 675 | 552 | 308 |
| *Symphyotrichum leave* (1) | 896* | 504 | 432 | x | 504 | 432 |
| *Taraxacum officinale* (4) | 621–622 | 522 | 402 | x | 522 | 440 |
| *Thalictrum venulosum* (4) | 746-748 | 609–615 | 469 | 752 | 610 | 470 |
| *Vicia americana* (4) | x | 522 | 179 | x | 522 | 179 |
| *Viola adunca* (5) | 406–407/397–398 | 583 | 443 | 412 | 577 | 443 |

^a^Numbers in parentheses following species names are replicates for Metzler et al. (i.e., the current study). Replication varied for Taggart et al. (2011).

^b^x = region where amplification failed for a species; v = highly variable species (>15 bp) where a consistent and useful identifier for that region could not be found; * = fragment length that was found in only one replicate and could not be confirmed by a closely related species in the current or other published studies. Ranges are provided when variability was found for a specific fragment length. Lengths from multiple binding sites are separated by a forward slash (/).

^c^Cells colored green in Metzler et al. indicate values that are within 1 bp of those in Taggart et al. (2011); red cells indicate values that are greater than 1 bp. Note that the *trnT-trnL* intergenic spacer was amplified using a modified primer in the current study and, consequently, will be 5 bp shorter than fragments amplified using Taberlet et al. (1991) primers A and B (see Table 2).
